# Novel phylogenetic methods are needed for understanding gene function in the era of mega-scale genome sequencing

**DOI:** 10.1093/nar/gkz1241

**Published:** 2020-01-16

**Authors:** László G Nagy, Zsolt Merényi, Botond Hegedüs, Balázs Bálint

**Affiliations:** Synthetic and Systems Biology Unit, Institute of Biochemistry, Biological Research Centre, Temesvari krt 62. Szeged 6726, Hungary

## Abstract

Ongoing large-scale genome sequencing projects are forecasting a data deluge that will almost certainly overwhelm current analytical capabilities of evolutionary genomics. In contrast to population genomics, there are no standardized methods in evolutionary genomics for extracting evolutionary and functional (e.g. gene-trait association) signal from genomic data. Here, we examine how current practices of multi-species comparative genomics perform in this aspect and point out that many genomic datasets are under-utilized due to the lack of powerful methodologies. As a result, many current analyses emphasize gene families for which some functional data is already available, resulting in a growing gap between functionally well-characterized genes/organisms and the universe of unknowns. This leaves unknown genes on the ‘dark side’ of genomes, a problem that will not be mitigated by sequencing more and more genomes, unless we develop tools to infer functional hypotheses for unknown genes in a systematic manner. We provide an inventory of recently developed methods capable of predicting gene-gene and gene-trait associations based on comparative data, then argue that realizing the full potential of whole genome datasets requires the integration of phylogenetic comparative methods into genomics, a rich but underutilized toolbox for looking into the past.

## INTRODUCTION

The post genomic era has brought about an exponential increase in the number of sequenced genomes, which has virtually eliminated sequence data being the limiting factor in comparative and evolutionary genomics. Currently, there are >200 000 genomes in GenBank (as of 15 July 2019, including nuclear and mitochondrial) and, although prokaryotic and fungal genomes dominate the landscape, plenty of genomes are available for all main lineages. In addition, data are coming along for lesser known or underrepresented phyla as well (1–4), especially with the spread of single-cell genomics (5). A whole new level of genomic data deluge is on the horizon with the launch of several large-scale genome sequencing projects, including ones aiming to sequence all living organisms on Earth (6) (Earth Biogenome Project) or in the UK (Darwin Tree of Life Project) and others specifically focused on major lineages such as plants (7–9), fungi (10,11) (1000 Fungal Genomes Project, 1KFG), vertebrates (12,13) (Genome 10K), birds (14) (Bird 10 000 genomes, B10K) or insects (15–17) (Insect 5000 Genomes, I5k), among others. Of these projects, the 1000 Fungal genomes project has been the first to break the 1000 genome boundary, as shown by the phylogenetically diverse collection of fungal genomes hosted by MycoCosm (18,19). All these data mean the foundations of comparative and evolutionary genomics, and the data flood we are about to see makes it timely to revisit some broad considerations of how all these data may and/or ought to be analyzed.

The completion of reference genomes for the main model species and decreasing sequencing prices led to the birth of comparative evolutionary genomics or phylogenomics (20), although the latter is more often used in the context of genome-scale inference of phylogenies. Comparative evolutionary genomics became one of the most rapidly expanding fields in biology, that seeks to explain evolved differences between species by using genomic data. Questions that comparative evolutionary genomics seeks to answer range from uncovering the phylogenetic relationships among species, understanding the evolution of genetic elements (e.g. genes, non-coding regions, etc.), how they affect organismal traits or, what genomic changes underlie the evolution of a phenotypic trait (21). The latter question is most promising from the perspective of relating unknown genes to traits of interest and for finding new genes that can fuel applications in biotechnology, agriculture or medicine. This article is focusing on the field of genomics that aims to relate observed genetic differences to phenotypes and evolutionary adaptations (e.g. metabolic capabilities, morphological structures, etc.) by systematic comparisons of whole genome sequences.

## ARE WE FULFILLING THE PROMISES OF COMPARATIVE GENOMICS?

Genome sequencing, by cracking the code of life is viewed by many as the ultimate key to decoding species’ biology and for better harnessing the diversity of life for basic theoretical, practical (biotechnological, medical) and societal challenges. The completion of the human genome and the avenues it opened were awaited with great excitement, for applications ranging from basic research to personalized medicine (22–24). Promises at the time were coming in numbers, but foreseen benefits to healthcare remained elusive for several years (25–27), leading some to ponder whether the project fulfilled its promises and even whether genomics was more hype than substance (25,28–29). It was only several years later that the human genome's broad impact, especially on basic research, became widely appreciated. While a genome's information content is certainly high, extracting signals from primary sequence is the real challenge and becomes increasingly so nowadays as the number of sequenced species grow exponentially (30).

The promises of comparative genomics are also ambitious, from understanding organismal biology and evolution, to explaining climate change (6), disease (31) or improving biotechnology (11,19) and agriculture (15,17), to name a few. Duly, the interest these promises and projects are sparking is intense. Indeed, combining genomes and phenotypic traits can, in theory, make it possible to answer questions that were only tractable in model species (32) before and will allow us to generate functional hypotheses for genes of the countless numbers of non-model species. This should eventually lead to closing the gap between the increasingly rapid accumulation of genomic sequences and the huge backlog of linking loci to phenotypes. It should also help inferring functional hypotheses to the vast regions of the protein space that are currently functionally uncharacterized (33–35). Given the potentials of large-scale genomics and the theoretical advances, we here present a personal evaluation of how current practices perform in achieving the promises and how we could do better. We posit that current practices under-utilize genomic information and offer suggestions on how to improve evolutionary and functional inferences from whole genomes.

### Known unknowns and unknown unknowns

A fundamental question for comparative genomics is which genomic loci underlie a given organismal trait (21,36). Identifying such genes without prior information is challenging, but comparing genomes of species that have the trait, to those that lack it should, in principle, make it possible. This is one of the great promises of comparative genomics but how much of this is realized?

Among the focal genes of comparative analyses, we distinguish two categories, based on what prior information is available for gene and trait. Genes whose association with the trait is supported by some prior information and we suspect are important for its evolution are referred to as known unknowns. Evidence may be coming from forward or reverse genetics in model species, from RNA-seq studies, functional annotations or many other sources. For example, a recent study (37) analyzed cytochrome p450 copy numbers in the koala genome in search for dietary adaptations to a highly toxic eucalypt-based diet. The choice of cytochrome p450 superfamily for scrutiny is based on its known role in detoxification (38), which guided the authors' choice in analyzing this superfamily. The study did not delve into genes that were *a priori* not known to be linked to detoxification but may be serving that purpose in the koala genome, potentially missing key gene families. In fungal genomics, carbohydrate-active enzymes (CAZy) are some of the most frequently analyzed gene families, comprising hundreds of individual genes in fungal genomes. They are known key players of wood-decay, a biotechnologically relevant trait (e.g. in biofuel production). Therefore, CAZy genes were the first candidates to be analyzed for understanding what genes differentiate efficient and weak wood-decaying fungi from each other (39). They eventually ‘made a big career’ in fungal comparative genomics, partly because they are the workhorse enzymes in wood-decay, but also because they are known players of the game. Their repertoires in fungal genomes indeed correlate with the species’ ability to decay wood, but are they the complete story or only a fraction of the big picture? Other gene families are also certainly needed for the complex process of wood decay, but these have not received even a fraction of the attention CAZymes received, because they are not known and are hard to crack.

Cytochrome p450s and CAZymes are ‘low hanging fruit’ for studies of koala dietary adaptations and fungal wood-decay, respectively, because we can link them to detoxification and lignocellulose degradation based on prior studies. They are known unknowns: while their analyses can be insightful, both wood-decay and digestion of toxic plants are sufficiently complex and understudied traits that we can assume with confidence that many other genes are also involved. We refer to the latter genes as unknown unknowns: they are linked to the trait, but we have no prior information on that. Unknown unknowns, might have generic functional annotation (e.g. conserved domains or gene ontology terms), but, from the perspective of the trait, are completely unknown and finding them should be an endeavor for comparative genomics studies.

### Unduly emphasis on known unknowns

For many evolutionary genomics studies the choice of genomic regions to be analyzed is influenced by prior knowledge, i.e. they are focusing on known unknowns. This is usually guided by interest in traits for which information is available from model organisms or some other source. This strategy will yield information on *a priori* selected sets of genes, in contrast to an unbiased screen which may find unknown unknowns that show a stronger link to the phenotype, but are not known yet. Restricting focus on known unknowns, thus, under-utilizes sequenced genomes and leaves some of the signal in the data untapped. This is not optimal, as combining genomes with traits has the potential to highlight unknown unknowns even in species or clades that are not amenable to laboratory experimentation (21). While new genomes are being published at an unprecedented rate, very few studies report on unknown unknowns, which results in an over-representation of already known genes in genomic studies. Some families get analyzed over and over again (see CAZy example for fungi, or a small subset of human genes (40)), whereas others remain on the ‘dark side’ of genomes, receiving no attention at all. This trend does not help closing the gap between genes of known and unknown function as more and more genomes become available and is in contrast with some of the basic goals of evolutionary genomics.

### How many unknown unknowns are there?

Assessing the number of unknown unknowns from a trait's perspective is challenging because there is an unknown number of genes underlying any given phenotypic trait. However, it is easy to provide an estimate from the perspective of genes of unknown functions (GUFs). Sequenced genomes usually contain a considerable fraction of genes whose function is unknown or that cannot be assigned any functional (e.g. pfam or GO) annotations. Such genes have proven the richest source of discovery of new protein folds and families (35).

**Figure 1.**
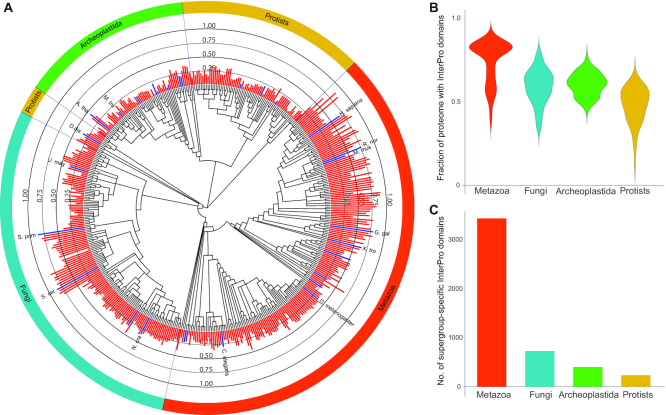
GUFs make up a significant proportion of eukaryotic genomes. (**A**) The proportion of protein coding genes across genomes of 461 species that can be annotated with approximate functions via 1-to-1 orthology to manually curated genes, based on the assumption that clear 1-to-1 orthology relationships are indicative of conserved and shared function. Orthology information and the tree were taken from the Orthologous MAtrix database (41). Tree was subsequently manually resolved to supergroups based on Deutekom *et al.* (121). Model systems from which functional information was propagated by orthology are marked with blue bars and the names of the most important ones are shown. Abbreviations as follows: H.sapiens—*Homo sapiens*, R.nor—*Rattus norvegicus*, M.mus—*Mus musculus*, G.gal—*Gallus gallus*, X.tro—*Xenopus tropicalis*, D.melanogaster—*Drosophila melanogaster*, C.elegans—*Caenorhabditis elegans*, N.cra—*Neurospora crassa*, S.cer—*Saccharomyces cerevisiae*, S.pom—*Schizosaccharomyces pombe*, U.may—*Ustilago maydis*, D.dis—*Dictyostelium discoideum*, A.tha—*Arabidopsis thaliana*, M.tru—*Medicago truncatula*. (**B**) Mean proportions of genes that contain known conserved domains from the InterPro database broken down by major eukaryotic group. Genomes were annotated with InterPro domains using InterProScan 77.0 (only the Pfam and CDD databases were considered, ignoring repeats and Domains of unknown Function, *E*-value cutoff 10^−5^). (**C**) The number of lineage-specific InterPro terms in the Metazoa, Fungi, Archeoplastida and Protist groups (collectively). Lineage specificity was assessed based on the array of species in which a given InterPro term was found.

A broad group of GUFs are those that lack any kind of functional annotation. Such genes are often just termed ‘hypothetical protein’ in genome annotations, because it is impossible to annotate them based on similarity to functionally characterized genes from other organisms. We assessed the number of GUFs based on InterPro domain contents in 573 eukaryotic genomes (Figure 1A). The proportion of genes without known InterPro domains is lowest for metazoans (13–61% per genome) and highest in protist lineages, where 35–87% of predicted genes have no functional annotations at all. Fungi (20–68%) and plants (24–54%) are intermediate, yet >30% of the genes in any genome are GUFs. Although some of such genes might be prediction errors, most are conserved across multiple species, highlighting the need for systematic approaches to discovering gene function. This is a particularly pressing need in less studied lineages such as protists or fungi (except yeasts), whereas the situation looks brighter for metazoans, which have traditionally been in the spotlight. In line with this, the number of lineage-specific InterPro annotation terms (i.e. those only known in a given kingdom) are highest in animals, but lowest in protists and fungi (Figure 1B), likely reflecting the amount of effort made to catalog conserved domain signatures rather than true differences in the number of conserved domains in these lineages.

We obtained another estimate of GUFs from genes to which precise functional hypotheses can be propagated based on 1-to-1 orthology relationships. We analyzed these patterns in 461 eukaryote species and pairwise orthology obtained from the OMA database (41). Genes for which more or less precise function can be inferred based on comparisons of 1-to-1 orthologs clearly show an enrichment around intensely researched model species (Figure 1C), with the two highest peaks corresponding to yeasts and vertebrates. Up to 80% of the genes of non-model species in these groups can be annotated with orthology-based functions, as opposed to 41, 38, 50 and 43% in protists, Archeoplastida, filamentous fungi (i.e. non-yeasts), and non-vertebrate metazoans, respectively. These figures aptly reflect the biased distribution of functional information around model species and calls for approaches targeting non-model organisms. For example, in fungi most functional information is coming from yeast, which has a highly stripped genome (42) that represents basic eukaryote functions properly, but not necessarily does conserved traits of filamentous fungi. Therefore, although some model fungi are particularly well-studied, the space of gene functions across the entire fungal kingdom in general remains poorly known. A similar bias has been observed within individual organisms; for example, it was reported that most research concentrates on ca. 2000 of the 19 000 genes on the human genome (40).

These data reveal a large number of GUFs in any given eukaryotic genome, which is consistent with some previous reports (33) and calls for systematic efforts and the development of approaches for charting their functional landscape.

## UNKNOWN UNKNOWNS CAN BE IDENTIFIED USING PHYLOGENY-AWARE APPROACHES

There is a myriad of approaches for finding genes linked to a particular phenotype, from mutagenesis assays, deletion libraries, co-expression analyses (33) etc. In comparative genomics associations between traits and genetic variants are inferred by comparing groups of genomes with or without the trait. In population genomics, this is now routinely accomplished by genome-wide association studies (GWAS) (43,44), analyses of quantitative trait loci (21) and related methods. GWASs investigate the entire genome and systematically look for co-occurrence patterns of a genetic variant and a trait in sequenced individuals (45). Although powerful at the population level, GWAS cannot be applied to comparisons of related species (21) because it cannot account for phylogenetic relationships (although attempts exist for modeling within-population phylogenetic structure (46,47)). The phylogeny is a source of strong signal that can mislead non-phylogeny-aware statistics (48–50), but a method that is similarly powerful as GWASs has not yet been widely adopted in multi-species comparative genomics.

**Figure 2.**
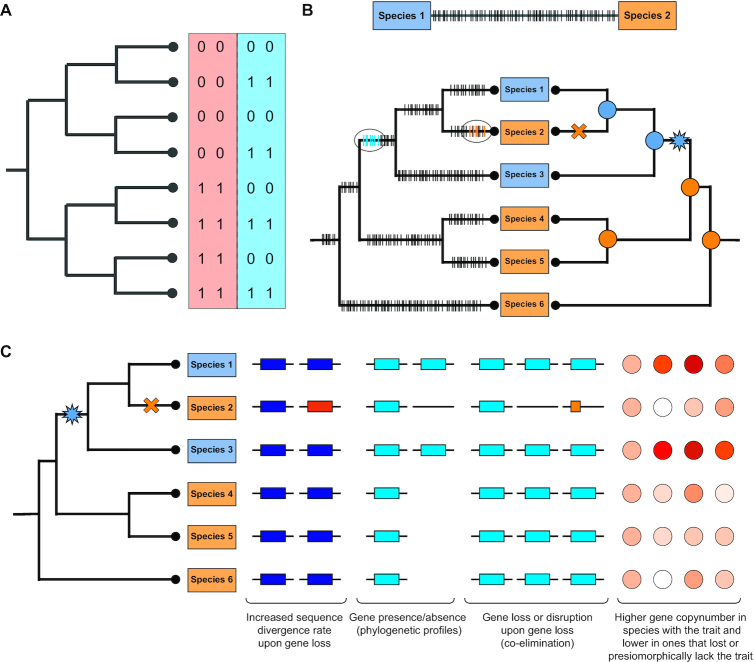
Basic principles of finding associations in genomics data. (**A**) A simple example illustrating the need of considering the phylogeny in comparative analyses of genomic data. Consider two pairs of genes (denoted by red and blue backgrounds) either present (‘1’) or absent (‘0’) in the eight species. Similarity in the presence/absence patterns of the first gene pair (red) are best explained by shared inheritance from the common ancestor. The second gene pair, on the other hand, can be explained by four coincident loss events along terminal branches, providing evidence for correlated evolution, and, thus potential functional linkage. Adapted from Barker *et al.* (49). Note that a similar logic works for detecting gene–phenotype associations. (**B**) Pairwise comparisons (upper panel) fail to adequately resolve the timing of genetic changes (marked by horizontal dashes), necessitating the use of phylogenetic methods which, on the other hand, can localize genetic changes to specific branches of the tree (bottom, left tree) and can narrow down the range of potentially relevant changes when compared with reconstructions of trait evolution (bottom, right tree). Trait distribution on the tree is shown by blue and orange rectangles and corresponding colored circles denote ancestral states. The gain and loss of the blue trait is denoted by a blue star and orange cross, respectively. (**C**) Some of the signal types that can inform analyses of gene– trait association. See text for explanation.

What options do we have for finding unknown unknowns in comparative studies of several species? The situation is quite simple in prokaryotes where gene presence/absence correlates well with traits (e.g. metabolic capabilities) and phylogeny may not strongly interfere with the analysis due to rampant HGT across species. For those situations, modified phylogenetic profiling methods could be used (Table 1). Phylogenetic profiling (51,52) was designed to find gene–gene co-occurrence patterns in a panel of species, as a way to identify functional gene modules and propagate functional annotations from one gene to the other (53). Although designed to find gene-gene associations, the original algorithm could easily be adapted to find gene-trait associations (see e.g. (54)). However, phylogenetic profiling does not consider the phylogenetic relationships of the species (despite its name), for which it has been criticized and shown to perform inferior to truly phylogenetic methods (49–50,55–56) (see Figure 2A). Barker and Pagel (49,50) showed that accounting for phylogenetic non-independence in the data using continuous-time Markov models in a maximum likelihood framework significantly improves the detection of gene-gene associations, especially if the model constrains genes to behave like Dollo characters (49).

**Table 1.** Methods for finding associations between genomic features based on macroevolutionary comparative data

Method	Type of association detected	Principle	Strength	Limitation	Refs.
Phylogenetic profiling	Gene–gene	Co-occurrence of genes across a panel of species	Simple, fast, widespread method	Cannot correct for phylogeny; cannot consider gene copynumber (only presence/absence)	(52,53)
CLIME	Gene–gene	Partitions genes across a panel of species into groups sharing a similar evolutionary history	Phylogeny aware; statistically sound and sensitive (incorporates tree HMMs); adaptable to phenotypes with modification	Cannot consider gene copynumber (only presence/absence)	(90)
Barker *et al.* 2007	Gene–gene	Identifies correlated gain/loss patterns in 1-to-1 orthogroups	Phylogeny aware; statistically well founded; adaptable to phenotypes with modification	Cannot consider gene copynumber (only presence/absence); the applied Markov models work poorly for Dollo-like characters (e.g. genes)	(49,50)
‘Forward genomics’	Gene–phenotype	Divergence from an ancestral sequence and co-elimination of genes in trait-preserving versus trait-loss species	Phylogeny aware; high sensitivity and specificity	Cannot consider gene copynumber (only presence/absence); only considers losses	(83,89)
REforge	*Cis*-regulatory sequence–phenotype	Same as ‘forward genomics’, for transcription factor binding sites	Phylogeny aware; considers *cis*-regulatory sequences; high sensitivity and specificity	Only considers losses; needs prior TFBS information	(88)
COMPARE	Gene–phenotype	Identifies shifts in gene duplication and loss rates upon the emergence and loss of a trait, respectively	Can analyze multigene families; integrates trait gain and loss information	Only considers gene duplications/losses (adaptable to other data types);	(42,45)
Chikina *et al.*	Gene–phenotype	Contrasts the rate of sequence evolution in trait-preserving versus trait-loss species	Phylogeny aware; can consider sequence-level divergence	Limited to 1-to-1 orthologs; only considers losses	(84,117)

All methods except phylogenetic profiling are computationally intensive, computational complexity is therefore not listed among the limitations.

The more severe limitation of phylogenetic profiling for eukaryotic genomics is that it considers simple presence/absence profiles of gene families (Table 1), which is problematic because eukaryotic gene families have intricate duplication/loss histories (55,57–58). The application of phylogenetic profiling to eukaryotes has, therefore, been limited (56,59), although examples of successful applications exist, especially for simple, single-copy gene families, like metabolic gene clusters (60,61) or in the context of strict 1-to-1 orthologs (54).

Finding links between genetics and trait has to consider three factors: the trait values of the compared species, the evolutionary history of each of the genetic elements (e.g. gene families) available for the analysis and the species phylogeny (Figure 2A and B). Parsimony- or likelihood-based methods (62,63) can be used to map genetic changes and trait gains/losses onto the phylogeny, followed by assessing the level of correlation between mapping of the trait and that for each of the genetic elements. Of the myriad types of genetic changes, gene family origins, sequence divergence, gene presence/absence and gene duplication and loss patterns are most commonly analyzed (Figure 2C), probably because these can be inferred from organismal gene catalogs relatively easily (see recent reviews (64–66)). In a comparative perspective, however, the logic of mapping duplications/losses can be extended easily to any type of genetic element in which homology relations (i.e. orthology, paralogy) can be established and its evolutionary history be reconstructed. Such mappings (67) provide information on the timing, temporal and taxonomic distribution of genetic changes and can be mined for various attributes, such as associations with trait evolution. It can also be used to infer which gene families show the highest duplication/loss rate across the trees, in specific clades or branches of the tree.

### Trait gain/loss signal informs searches for unknown unknowns

Theory dictates that, of the thousands of genes and non-coding elements in a genome, the ones that are linked to a trait should show correlated evolutionary changes with it. That is, the gain or loss of the trait should correlate with gains or losses of genetic elements, which is identifiable when viewed across a panel of species. The ideal, ‘textbook’ case involves a single change, such as a gene duplication or a single nucleotide polymorphism that is necessary and sufficient for a new trait to evolve. This is probably rarely the case (although examples exist (68)), however, and an array of changes are probably more often necessary for the emergence of the trait. Many of these changes might predate the trait in evolutionary time, whereas a single or a few might directly lead to its manifestation as a phenotype (cf. threshold model (69)). It should be noted that the gain of the trait may also correlate with a range of genetic changes that are induced by, rather than being causative of, its emergence, making it hard to distinguish correlation from causation.

Using this logic, genetic changes that correlate with the emergence of a trait can be identified in genome-wide catalogs of genetic innovations. Several methods have been proposed for this (see below and Table 1), although, as may be expected in new fields, no single method has yet been applied more than a few times. To inform our search, we should understand the phylogenetic relationships of the species being compared and we should know in which node(s) along this phylogeny the trait showed changes. Ancestral character state reconstructions can be used to map gain(s) and loss(es) of the trait onto the phylogeny and obtain a view of character state transformations (see recent reviews (70–72)). From the perspective of the genetics, a range of signals may be analyzed, including changes in selection regime, *de novo* gene (family) birth, gene duplication/loss, rearrangements, rewiring, SNPs, regulatory networks, splicing and expression patterns (73), epigenetics, etc. In the simplest approach, one may ask if the group of species with the trait (including their ancestral nodes) are enriched for certain genes relative to those not having the trait, given the phylogeny. Another simple approach is asking what gene families (orthogroups) are gained/lost in parts of the tree where the trait is gained or lost. Several example studies (42,54,74–75) and dedicated pipelines (76,77) testify the validity of these approaches, which is, however, conditioned on the correct identification of strict orthogroups and on simple gene presence-absence being a good predictor of trait evolution. This is often not the case, as evident, for example, from the deep conservation of multicellularity-related genes in many clades (78,79). A higher resolution approach considers not only gene family origin and loss, but also gene duplication and loss (80), providing a fine-grained view on genetic changes in relation to changes in trait values (see below).

Because we are looking for correlation, the more character state transitions the trait shows across the phylogeny, the more precise our search can get (81). A trait with a single gain on the tree allows us to identify genetic changes that are coincident with its emergence, which potentially yields a long list of genes, among which we cannot differentiate further, unless we have other types of data. A trait with two state transitions (e.g. a gain and a loss) gives us more precision, by allowing us to identify genes that change in both cases when the trait does and may be viewed as the minimum number of state changes necessary to find gene–phenotype associations. Trait loss, in particular, convergent loss, can provide valuable information for identifying linked genetics because relaxed selection on the genetics of the lost trait leads to divergence and/or complete elimination of the underlying genetic elements (recently termed co-elimination of genes (82)).

Datasets with convergent trait losses are particularly signal-rich, because losses happen to a homologous genetic background and therefore genetic changes are likely shared by convergent loss events. Several studies exploited convergent losses to pinpoint genes linked to the trait. Hiller *et al.* (83) devised an approach to find genes, across all alignable genomic loci, with an increased rate of sequence divergence in species that lost a given phenotype (in the study, the ability to synthesize vitamin C) (see also an alternative method (84)). The method has been extended to the complete loss of genes (85,86) and to screening divergence in transcription factor binding sites (87,88) in relation to trait loss, the latter vividly demonstrating that the logic is not only applicable to genes, but also other genetic elements. Phylogenetically corrected versions of the method (utilizing phylogenetic generalized least squares) have also been developed and used to find vision-related genes in a dataset that contains two independently evolved subterranean mammals (89). Because the logic of these approaches is similar to that of forward genetics, the term ‘forward genomics’ has been coined (83) and it has been argued that collections of phenotypic data would allow many-to-many analyses of trait—gene association.

CLIME (90) is a Bayesian tool that considers the species tree topology and gene gain and losses to infer groups of genes that share the same evolutionary history (described by tree HMM-s). Such groups may represent functional modules or pathways (evolutionarily conserved modules) and can be used to infer putative functions for unknown genes. CLIME can be considered a sophisticated model-based and phylogeny aware phylogenetic profiling algorithm. It was reported to perform better on presence/absence matrix of homologs than on strict 1-to-1 ortholog matrices. The simultaneous inference of optimal gene partitioning scheme and parameters of HMMs describing the evolutionary history of the genes in one Bayesian MCMC framework is attractive and yields high statistical power. CLIME was found to perform well on genes with ≧6 losses and moderately well on ones with ≧4 losses (90).

Considering gene duplication/loss rates instead of gene presence/absence can further improve predictions. The COMPARE (42,55) pipeline integrates signals of gene duplication and loss with trait gain and loss for predicting gene family—trait associations. It uses gene trees to infer gene duplication/loss events (based on the species (91,92) overlap principle), taking into account the complex one-to-many orthology/paralogy relationships (64–65,91,93) characteristic of eukaryotic gene families. Inferred duplications and losses are mapped onto the phylogenetic species tree, yielding reconstructed ancestral genomes and fully resolved gene duplication and loss histories across clades and through time. This can be analyzed using comparative methods or mined for gene families that show elevated duplication rate in part(s) of the tree where a trait is gained and/or elevated loss rates where it is lost (55). This method has been used to infer the tempo and mode of genome evolution through time and across clades (94–96), to reconstruct genetic innovations underpinning the evolution of multicellular fungi (78,97), that of the convergent origins of yeasts (42) and to make predictions on the genetic bases of efficient wood-decay strategies by fungi (55). This latter exercise was aimed to find unknown unknowns of the genetics of wood-decay. The search returned 409 gene families which, as expected, contained several CAZyme families that were previously reported to be associated with wood-decay (i.e. known unknowns) but also hundreds of novel families. A comparison to three gene expression datasets showed that >60% of these families were also significantly upregulated when wood was the single carbon source in the experiment, providing independent validation for the predictions. It should be noted that wood-decay represents a fitting trait for this approach, because it evolved once in Agaricomycetes fungi and was lost several times (seven losses in the dataset). Nonetheless, simulations showed that COMPARE had high precision in detecting gene–trait associations even for traits with a single gain and a single loss (55). This highlights the power of using gene duplication/loss rates for understanding genome evolution, which comes at a high-computational cost, however, and the validity of the findings is conditional on accurate gene family assignments (i.e. orthology and paralogy detection).

Finding unknown unknowns can also be a daunting task and sometimes will not yield sensible results. A search for gene families that fit the phylogenetic pattern of nitrogen-fixing symbioses of plants in a dataset of 37 genomes failed to find any positive hits (98). However, it turned out this was because the evolutionary history of nitrogen-fixing symbioses is not accurately described by the phylogenetically most parsimonious scenario (single gain, multiple losses), which eventually suggested a mechanism of trait evolution that cannot be expected to fit into a search strategy like that (99).

These examples show the wide range of approaches that can be used to identify unknown unknowns using comparative genomics. The methods are available, though there is not a long record of their application in evolutionary genomics, a status that will hopefully change in the near future. A common feature of all these is the explicit modeling of phylogenetic relationships that allows the analysis to distinguish between similarity caused by common descent from that caused by similar selective pressures (49). Phylogenetic comparative methods have, for decades, been developed for these very situations, although mostly in isolation from genomics. We next argue that better integration of phylogenetic methods will empower us to answer more exciting questions in evolutionary genomics.

### Phylogenetic comparative methods are a rich toolbox for genomics

Phylogenetic comparative methods are statistical approaches that combine information on species relatedness with contemporary trait values to infer historical patterns of evolution (48,71). We will not go into details about methods (only refer to recent reviews), just note that there are elaborate models for analyzing various attributes of the evolution of discrete or continuous characters, which could be transplanted into genomics to answer new types of questions on genome or trait evolution. We offer a few examples and note in parentheses the potential applications in the context of genomics. Without attempting to be exhaustive, established methods exist for analyzing gains and losses of character states (48,100–101) (e.g. presence/absence of a genetic element, expression level) across the phylogeny, inferring changes in trait values (e.g. expression values) (102) along trees, the distribution of character state changes across time or clades (71,103) (e.g. assessing rates of genetic evolution through time), for assessing variation in the timing of tree branching events (104–107) (which, in gene trees mean duplications) or for reconstructing ancestral character states (many of these have been adopted in ancestral gene content/gene order/gene sequence reconstructions) (67,83,88–89,108–111). We argue that these methods represent a largely unexploited methodological resource for genomics and that they could be used to extract valuable signal from genomic datasets. One aspect that was quickly adopted by genomicists are parsimony-based methods for inferring the placement of gene duplications/losses or other genetic events (112,113), ancestral genomes (109) or gene order. Dollo parsimony is especially well-suited to genetic data (62) and is conceptually easy to grasp. However, there is a suite of other methods that could be deployed in comparative genomics. For example, by applying the Binary-state speciation and extinction (BISSE) model (114), which measures a binary trait's effect on lineage diversification, to gene trees, it was possible to show that class-II-peroxidases (which degrade lignin in plant tissues) show a significantly higher gene duplication rate in efficient wood-decay fungi than in weaker or non-decayers (39). Although class-II-peroxidases were known before as key players of wood-decay, this analysis is proof-of-concept that phylogenetic comparative methods can be used in novel ways to extract valuable signal from genomic datasets. Another attractive approach for analyzing the genetics of a trait would be assuming a gradual assembly of a genetic toolkit in a way similar to what the threshold model for phylogenetic comparative analyses implements (69,115). In that model, the discrete (presence/absence) trait we observe is the function of a continuous underlying quantity (liability (69), e.g. genetic innovations) that, if builds up to a sufficient level, allows the trait to manifest. One can envision applications of this model to complex multigenic traits, where genetic innovations can be expected to come along in a gradual manner, making traditional analytical methods inadequate. These examples illustrate, along with several others above, that phylogenetics offers a largely untapped pool of tools for evolutionary genomics.

### Computational challenges

A significant question is how current methods scale with the number of genomes analyzed and whether we will be able to deal with the flood of data expected in the coming years. Most of the pipelines discussed above (55,83–84,90) start with computationally intensive steps (all-vs-all searches, orthology, gene tree and species tree inference or combinations of these) and thus how these scale with the number of genomes analyzed determines the overall computational burden of the analysis. To date, phenotype–genotype associations have been analyzed in datasets comprising up to 62 or 100 mammalian species by forward genomics (116,117), and up to 62 and 117 fungal genomes by COMPARE (55) (Miyauchi *et al.* in preparation). Run times for large analyses can be prohibitive and require high-performance computing facilities. Fast methods that allow similarity searches and orthology inference in thousands of genomes without compromising accuracy are now becoming available (see e.g. (118,119)) and preliminary analyses using these suggest that analyses of up to 1000 genomes are feasible (Balint *et al.*, unpublished data). Similarly, the need to infer species trees could soon be bypassed as large-scale genomic trees, from which topologies for subsets of species can be extracted, become increasingly available.

A promising strategy for bypassing the need of re-running some of the computationally intensive steps is the integration and periodic updating of results into openly available and searchable databases. These could be mined for associations with any trait that shows state transitions in the suite of species represented and could provide an open platform for predicting gene function based on phylogenomics.

## CONCLUSION

Exploring complex datasets is a prime challenge in today's biology. Much of genomics currently is explorative—that is, we let the data guide us toward interesting patterns—and the more efficiently this happens, the more efficiently we get to discoveries and can generate hypotheses. Therefore, creativity in data analysis needs to be emphasized and enhancing signal extraction from currently available genomics data should be a priority. We argued above that for evolutionary genomics, phylogenetic comparative methods provide a rich and under-exploited toolbox that evolutionary biologists have been developing for many decades. Evolutionary genomics could build on that or other approaches (e.g. artificial intelligence methods (120)) to extract signal and make informed predictions on gene–phenotype associations, genome evolution or the principles of evolutionary adaptation from genome-scale data.

While some would probably argue that increasing the size of genomics datasets, better integration of different data types (e.g. genomic, transcriptomic, proteomic), higher resolution (e.g. single-cell) or more reference-quality assemblies (6) is the way to go in evolutionary genomics, our standpoint is that better approaches for analyzing the data at hand and the extension of current methods to utilize multiple evolutionary signals in comparative datasets (gene duplications/losses, parallel amino acid changes, positive selection) are of utmost importance. This is not only because these would allow making biological inferences from data already at hand, but also because better extraction of signal from genomic data can provide functional, testable hypotheses and can drive -omics science toward a more hypothesis-driven state.
